# Factors Affecting Handgrip Strength in Menopausal Women at High Risk of Sarcopenia: A National Population-Based Study

**DOI:** 10.3390/healthcare12242590

**Published:** 2024-12-23

**Authors:** Anna Lee, Sooyeon Park

**Affiliations:** 1 College of Nursing, Yonsei University, 50-1, Yonsei-ro, Seodaemun-gu, Seoul 03722, Republic of Korea; annalee@yuhs.ac.kr; 2 Mo-Im Kim Nursing Research Institute, Yonsei University, 50-1, Yonsei-ro, Seodaemun-gu, Seoul 03722, Republic of Korea; 3 College of Nursing, Konyang University, 158 Gwanjeodong-ro, Seo-gu, Daejeon 35365, Republic of Korea

**Keywords:** hand grip strength, menopause, muscle strength, sarcopenia

## Abstract

**Background/Objectives**: Hand grip strength (HGS) reflects muscle strength and is an important indicator of sarcopenia. There is a gap in the research regarding the determinants of relative HGS that take sex differences into account. Therefore, this study aimed to evaluate the association between relative HGS and physical, behavioral, and psychological factors in menopausal women from South Korea. **Methods**: This study used data obtained from the 7th National Health and Nutrition Examination Survey (2016–2018), which had a complex, stratified, and multistage sample design. A total of 2129 menopausal women aged between 40 and 64 were included in this cross-sectional study. To evaluate physical, behavioral, and psychological factors associated with relative HGS, a multiple regression analysis was used. **Results**: In this study, the sociodemographic factors that influenced relative HGS were education (high school: B = 0.03, *p* = 0.001; college: B = 0.04, *p* = 0.003) and marital status (live with: B = 0.04, *p* = 0.004). Among the physical factors, abdominal obesity (B = −0.16, *p* < 0.001) and comorbidities (B = −0.03, *p* = 0.026) were associated with a significantly lower relative HGS. Regarding behavioral factors, relative HGS was significantly lower for those who slept more than 8 h (B = −0.03, *p* = 0.041) than for those who slept 6–8 h. Regarding psychological factors, relative HGS was significantly higher for those with fair (B = 0.04, *p* = 0.001) and good (B = 0.06, *p* < 0.001) self-rated health. **Conclusions**: Relative HGS is associated with physical, behavioral, and psychological factors in menopausal women. These findings can inform research and guidelines for sarcopenia prevention using relative HGS as an indicator of health status.

## 1. Introduction

After menopause, women face a higher risk of health challenges, including cardiovascular diseases and cancer [1,2,3]. Among many health problems, sarcopenia is a musculoskeletal disease characterized by a decrease in muscle strength or skeletal muscle mass [4] and decreased physical performance, and it is reported to occur earlier in women compared to men [5]. Generally, muscle mass and strength reach a maximum in early adulthood (31–40 years old) and decline thereafter [6]. Women are at a higher risk of sarcopenia as they age and undergo menopause [7]. In women, age alone cannot explain the decline in strength after the age of 50 years [8], and estrogen concentrations decrease during menopausal transition, contributing to a decline in bone and skeletal muscle function [9,10].

Hand grip strength (HGS) is an important muscle strength index for diagnosing sarcopenia [4]. Low HGS is an important health biomarker associated with overall mortality [11], and muscle strength measured by HGS is considered a more important indicator of sarcopenia than low muscle mass, as low HGS predicts accidents, fractures, and mortality in the entire population [12]. It is a noninvasive procedure, relatively inexpensive, and correlates well with other measures of physical function [6,13]. The measurement of muscle strength has been endorsed as the preferred method by professional sarcopenia organizations. HGS measurement is becoming increasingly common as a clinically feasible screening tool for determining muscle weakness and detecting other clinically relevant health outcomes [14]. As muscle strength gained attention as a biomarker of sarcopenia, the European Working Group on Sarcopenia in Older People revised the classification, with low muscle strength constituting “possible sarcopenia” and low muscle mass confirming the diagnosis in 2019 [4]. HGS has been identified as an important indicator for the early diagnosis of sarcopenia in community settings and is especially true for menopausal women who are at high risk of sarcopenia due to decreased female hormones [15].

HGS has been used as a measure of various health functions and is known to be influenced by both physical and psychological factors [16,17]. In addition to demographic factors such as age, sex, and socioeconomic status [18], HGS is influenced by physical factors, including obesity and comorbidities, lifestyle [19], and psychological factors, including depressive symptoms and self-rated health (SRH) assessment [11,16,20]. Given that HGS has been consistently reported as a useful screening tool for muscle strength, there is a need to further understand the various factors that influence HGS and their underlying associations in populations at risk of sarcopenia.

HGS has been evaluated as a reliable indicator [21] and is associated with significant public health concerns in that low HGS in older populations is strongly linked to adverse health outcomes, including frailty, falls, or increased mortality [22,23]. However, the relevance of muscle strength as a reliable marker of sarcopenia in middle-aged and pre-sarcopenia populations remains unclear [24]. Moreover, the multifactorial determinants of HGS in middle-aged menopausal women—a group at risk for sarcopenia—are not well defined due in part to the variability in measurement methods employed across studies [25]. Among the methods of measuring muscle strength, relative HGS standardized to body mass index (BMI) provides consistent results [26]. Meanwhile, the determinants of relative HGS, considering sex differences, have not been characterized.

The current study is based on the assumption that there are physical, behavioral and psychological factors that influence the HGS of menopausal women at high risk of sarcopenia. Therefore, this study aimed to investigate the multifactorial determinants of HGS using a nationwide population-based survey.

## 2. Materials and Methods

### 2.1. Study Design and Population

This secondary data analysis study sought to identify the influencing factors of relative HGS in menopausal women. It utilized the raw data of the 7th KNHANES (2016–2018), in which all HGSs were measured in the National Health and Nutrition Examination Survey provided by the Korea Disease Control and Prevention Agency (KDCA) [27]. The KNHANES is conducted annually at the national level and produces nationally representative and reliable statistics on the health status, health behaviors, and food and nutrition intake of the population.

The KNHANES sampling framework was based on the latest Population and Housing Census data at the time of the study design [27]. A two-stage stratified cluster sampling method was adopted, with enumeration districts and households as primary and secondary units, respectively [27]. The framework was stratified by city/province, urban/rural areas, and housing type (general housing, apartments), incorporating residential area proportions and household head education levels to improve representativeness [27].

**Figure 1:**
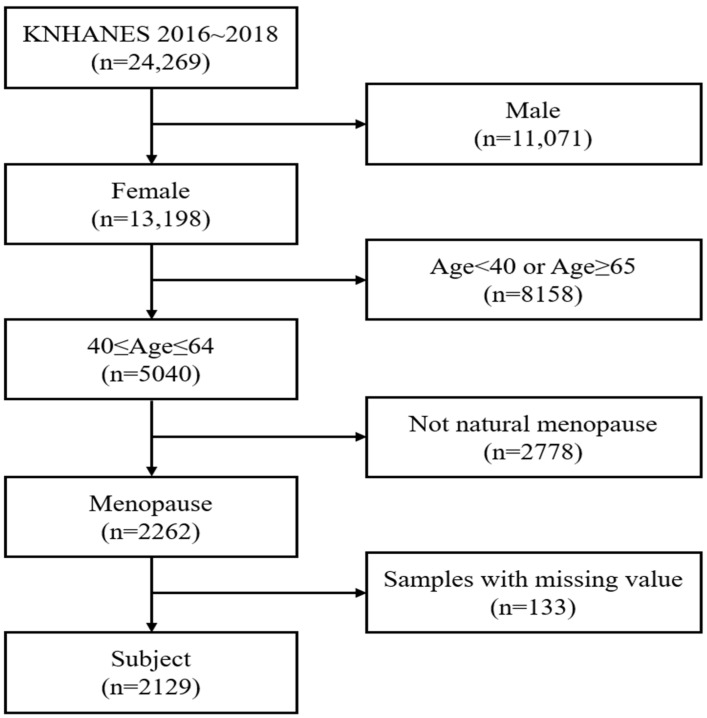
Flow chart of study participant selection.

Of 24,269 samples, 13,198 female samples were extracted, 5040 of whom were aged 40–64 years, and 2262 were women who had undergone natural menopause (i.e., cessation of menstruation for at least 12 consecutive months without other physiological or pathological causes). Women who had undergone artificial menopause (e.g., due to medical conditions or surgical interventions) were excluded to minimize confounding factors and ensure accurate study outcomes. Additionally, participants with missing data values for the main variables were excluded. Finally, 2129 women were included in this study (Figure 1).

### 2.2. Study Variables

#### 2.2.1. Relative Hand Grip Strength

The HGS test used a digital grip strength dynamometer (T.K.K.K 5401, Tokyo, Japan) with a 0.1 kg measurement unit. The participants stood with their backs and shoulders straight; their legs were spread approximately the width of their pelvis while lowering both arms. Without bending the elbows or wrists, grip strength was measured thrice for each hand. A maximum of six total HGS measurements for both hands were made. Relative HGS was calculated as the absolute HGS divided by BMI, as recommended previously [26,28].

#### 2.2.2. Multifactorial Factors

The evaluated variables included physical, behavioral, and psychological factors. The physical factors included BMI, abdominal obesity, and comorbidities. The BMI was classified as under “18.5 kg/m^2^”, “18.5~24.9 kg/m^2^”, and “25.0 kg/m^2^ and over” Abdominal obesity was defined as a waist circumference ≥ 85 cm. Comorbidities were categorized as “yes” or “no” based on whether a person was diagnosed with two or more of the following chronic diseases simultaneously: hypertension, dyslipidemia, stroke, myocardial infarction, angina, osteoporosis, and diabetes. Among the behavioral factors, sedentary time was classified into “more than 8 h” and “less than 8 h”. Sedentary behaviors were defined as those requiring 1.5 metabolic equivalents or less energy expenditure in a sitting, reclining, or lying position while awake [29]. According to the NHANES, these behaviors included sitting at a desk; sitting with friends; traveling by car, bus, or train; reading; writing; playing cards; watching television; playing games (e.g., Nintendo, computer, PlayStation); using the internet; listening to music; etc. Alcohol consumption was classified as “yes” or “no” based on whether or not they drank alcohol at least once per month, and sleep duration was classified as <6 h, >8 h, and 6–8 h. Of the psychological factors, depression symptoms were categorized as “yes” or “no” based on whether a person felt somewhat or severely depressed. Perceived stress was classified as “high” or “low” based on perceived stress levels.

#### 2.2.3. Sociodemographic Characteristics

Sociodemographic characteristics included age, household income, educational level, marital status, and economic activity. Age was classified into “40s”, “50s”, and “60s”, and household income was reclassified as “high”, “medium”, and “low”, based on income quartiles. Educational level was classified as “college graduate or higher”, “college graduate”, or “college graduate or lower”. Marital status was classified as “living with” or “living without” based on whether or not the spouses lived together. Economic activity was classified as “yes” or “no” according to their current job status.

### 2.3. Statistical Analysis

As this study utilized raw KNHANES data, a complex sample analysis was conducted to reflect the complex sample design by specifying the integrated weights, stratification variables, and clustering variables provided by the KDCA. The SPSS Statistics 26.0 (IBM Corp., Armonk, NY, USA) was used for data analysis, and statistical significance was determined at an alpha level of 0.05. Frequency and descriptive statistical analyses were conducted to identify the general characteristics and physical, behavioral, and psychological factors of menopausal women aged 40–64 years. Descriptive statistical analyses were conducted to determine the mean levels of the variables measured as continuous variables and relative HGS of the patients. Analysis of variance (ANOVA) using a general linear model was conducted to verify the differences in relative HGS according to the patients’ general characteristics and physical, behavioral, and psychological factors. Finally, a multiple regression analysis using a generalized linear model was conducted to verify the factors affecting the relative HGS of menopausal women.

## 3. Results

### 3.1. Sociodemographic and Multifactorial Characteristics of the Participants

The mean age was 57.05 ± 0.11 years, with the highest proportion of 50–59-year-olds (64.0%). Moreover, 55.5% of the respondents reported “medium” household incomes, and 42.5% reported “less than a high school” education level. Regarding marital status, 80.6% of the respondents were currently living with their spouses, and 58.4% were currently economically active.

Among the physical factors, the average BMI was 23.95 ± 0.09 kg/m^2^, with 65.3% in the 18.5–24.9 kg/m^2^ group. The average waist circumference was 80.49 ± 0.25 cm; approximately 27.7% had abdominal obesity (≥85 cm). Comorbidities were reported by 20.9%.

Regarding behavioral factors, sedentary time averaged 7.10 ± 0.08 h, with 56.8% reporting “less than 8 h” of sedentary time. Alcohol consumption was reported by 62.1% of current drinkers, and sleep duration averaged 6.99 ± 0.03 h, with 69.3% reporting 6–8 h.

Regarding psychological factors, 11.2% reported depressive symptoms, 24.0% reported high levels of perceived stress, and 55.7% rated their SRH as fair. For the dependent variable, absolute hand grip strength was 7.2% for those under 18 kg. Relative grip strength divided by BMI was 1.01 ± 0.01 (Table 1).

### 3.2. Relative Hand Grip Strength by Subjects’ Characteristics

In terms of sociodemographic characteristics, significant differences were observed in the relative HGS according to age (*p* < 0.001), household income (*p* < 0.001), education (*p* < 0.001), and marital status (*p* < 0.001). Relative HGS was significantly higher for those in their 40s and 50s than for those in their 60s. Moreover, it was significantly higher in the medium-income group than in the lower-income group and significantly higher in the high-income group than in the medium-income group. Relative HGS was also significantly higher for those with a high school degree or higher than for those with a middle school degree or lower, and was significantly higher among those living with their spouses (Table 2).

Regarding physical factors, significant differences were detected in relative HGS according to BMI (*p* < 0.001), abdominal obesity (*p* < 0.001), and comorbidities (*p* < 0.001). Relative HGS was higher in those with a BMI of 18.5–24.9 kg/m^2^ than in those with a BMI ≥ 25.0 kg/m^2^, in those with a BMI of <18.5 kg/m^2^ than in those with a BMI of 18.5–24.9 kg/m^2^, and in those without abdominal obesity or comorbidities (Table 2).

Relative HGS did not differ significantly based on any behavioral variables; however, the effect of sleep duration approached significance (*p* = 0.061).

Regarding psychological factors, a significant difference in relative HGS was observed based on SRH (*p* < 0.001). The results suggested that a better SRH was associated with a higher relative HGS (Table 2).

### 3.3. Factors of Relative Hand Grip Strength

To verify the factors affecting the relative HGS of menopausal women in this study, multiple regression analysis was conducted, including the variables that showed significant differences in relative HGS in the previous ANOVA (Table 3). BMI, a variable included in the formula for relative HGS (absolute HGS/BMI), was excluded as an independent variable. The sociodemographic variables, education and marital status significantly impacted relative HGS. Additionally, relative HGS was significantly higher for those with a high school diploma (B = 0.03, *p* = 0.001) and college degree or higher (B = 0.04, *p* = 0.003) than for those with a high school diploma or less, and significantly higher for those living with a spouse (B = 0.04, *p* = 0.004).

Regarding physical factors, abdominal obesity and comorbidities significantly affected relative HGS. Abdominal obesity was associated with significantly lower relative HGS (B = −0.16, *p* < 0.001), and comorbidities were associated with significantly lower relative HGS (B = −0.03, *p* = 0.026).

In behavioral factors, sleep duration had a significant effect on relative HGS, with significantly lower relative HGS among those with excessive sleep compared to those with adequate sleep (B = −0.03, *p* = 0.041).

Regarding psychological factors, SRH significantly influenced relative HGS, with those in fair (B = 0.04, *p* = 0.001) or good (B = 0.06, *p* < 0.001) health having significantly higher relative HGS than those with poor health.

**Figure 2:**
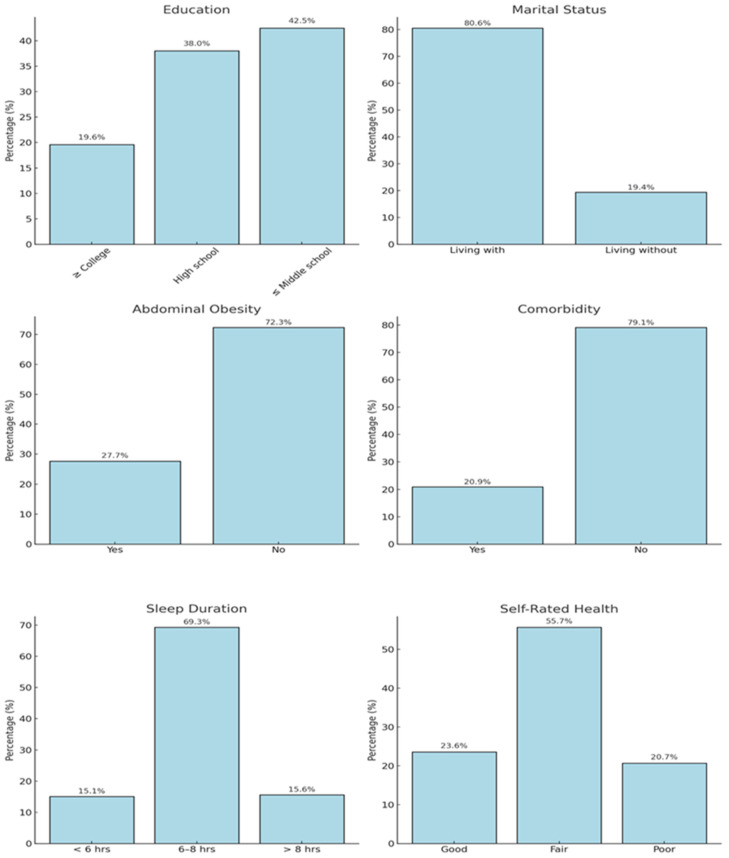
Percentage of major study variables.

As a result, we concluded that menopausal women with higher levels of education, living with a spouse, no abdominal obesity, no comorbidities, no excessive sleep, and good SRH had significantly higher relative HGS (Table 3). Figure 2 shows the percentage of major study variables from Table 3 that are significantly associated with relative HGS.

## 4. Discussion

The results of the current study identified various sociodemographic, physical, behavioral, and psychological variables that are significant predictors of relative HGS in menopausal women.

The prevalence of possible sarcopenia in menopausal women assessed by absolute HGS was reported as 7.2% by the Asian Working Group for Sarcopenia 2019 [30]. However, this was lower than the ~12% reported by a recent Asian study in postmenopausal women aged 45–65 years in the pre-sarcopenia stage [7]. A study on community-dwelling, middle-aged and older women in China, not postmenopausal women, reported a prevalence of 7.2% for possible sarcopenia using the same HGS criteria [31], consistent with the current study results. However, most previous studies that adopted the HGS criteria did not consider sex differences, while others included predominantly older women. Additionally, the diagnostic criteria, methods for assessing muscle strength, and cutoff points vary among studies, making accurate comparisons difficult. Further studies are needed to provide evidence on the prevalence of sarcopenia in menopausal women and the prevalence of relative HGS criteria.

Among the sociodemographic factors, education and marital status were significantly associated with relative HGS in this study, which is consistent with the findings of many studies [20,32,33]. The association between education and HGS in women is consistent with what has been reported in many studies. In a study of the association between socioeconomic status in early adulthood and middle age and HGS, education was associated with HGS in women but not in men [33]. The findings are also consistent with a previous systematic review of the association between HGS and demographic and lifestyle factors in the adult population, which found that among demographic factors, lower educational attainment was associated with lower HGS in women [19]. Another study found that physical activity was the main mediator of the association between education and HGS, with higher levels of education being associated with greater participation in physical activity and therefore greater HGS [34]. As education is a modifiable factor, increasing education as an influencer of HGS may be able to strengthen HGS.

Marital status was also significantly associated with HGS. Married adults living with a spouse had a lower risk of sarcopenia, consistent with the findings of a previous study on muscle strength [35]. Marital status exerts a protective effect on health and mortality by providing reciprocal care and acceptance, which is important for young and middle-aged individuals [36]. However, the opposite has been reported for men, with spouses leading to a lower HGS [11,37]. Hence, further exploration of sex differences in HGS and marital relatedness is warranted.

Regarding physical factors, waist circumference and comorbidities significantly influenced HGS. As previously reported [35,38], a higher waist circumference is significantly associated with lower HGS. However, abdominal obesity is a better indicator, as BMI includes lean muscle mass in its calculation, a determinant of HGS. In this study, HGS was highly associated with abdominal obesity [39]. A large waist circumference exacerbates the penetration of excess body fat into the muscles, reducing muscle quality and physical performance [4,40]. Additionally, abdominal fat can be a risk factor for sarcopenia due to its inflammatory effects, leading to chronic inflammation and greater muscle loss [41]. Waist circumference is a major risk factor for metabolic syndrome; HGS negatively correlates with metabolic syndrome [42]. Hence, addressing abdominal obesity as a factor influencing HGS is crucial. Furthermore, comorbidities were significantly associated with HGS, which is consistent with previous studies [43,44]. Systematic reviews and meta-analyses on sarcopenia and comorbidities have reported that cardiovascular disease, dementia, diabetes, and respiratory disease share common risk factors for sarcopenia, including physical inactivity and a sedentary lifestyle [44]. Similarly, pathophysiological factors, such as inflammation and oxidative stress [45], increase the risk of sarcopenia. Therefore, effective preventative and treatment strategies for sarcopenia in middle-aged menopausal women should focus on comorbidities.

Among the behavioral factors, sleep duration was significantly associated with HGS, with those who slept more than 8 h having lower HGS Similarly, a previous study reported that compared to those who slept less than 5 h daily, those who slept 6 to 7 h had a higher HGS, while those who slept 9 h or more had a lower HGS [46]. A recent systematic review reported that a higher HGS was associated with adequate sleep [47]. Moreover, in middle-aged and older adults, decreased muscle strength was significantly associated with poor sleep quality and insufficient sleep duration [48,49]. Indeed, skeletal muscle metabolism and function maintenance are linked to circadian rhythm [49]. Decreased sleep duration and quality, altered circadian rhythm, and sleep disorders promote protein degradation, alter body composition, and increase the risk of insulin resistance, all of which are associated with sarcopenia. Furthermore, the association between sleep duration and muscle strength differs significantly between males and females [50]. Short sleep durations are associated with lower HGS in males, whereas shorter and longer sleep durations are significantly associated with lower HGS in females [51]. Given that sleep is an essential component of modern lifestyles, it is important to consider sleep duration as a significant determinant of HGS in menopausal women and to further explore its relationship with HGS, including sleep quality.

Among the psychological factors, better SRH was associated with significantly higher HGS [52]. SRH is a multidimensional measure of health status, including health behaviors, which is subjectively assessed and depends on sociodemographic and physical health characteristics. In the present study, better SRH was associated with higher HGS, which agrees with previous study results [53,54]. However, SRH has been reported as a sex-specific construct, with only males showing significant differences in HGS [11], suggesting that further associations between HGS and SRH need to be explored. Hence, SRH differs between males and females, while perceptions influence the health profiles of males and females.

Considering that sarcopenia not only impacts older adults [35], is necessary to evaluate muscle strength loss and its relationship with health across all ages. Identifying the factors that influence HGS as a marker of sarcopenia is necessary to understand the potential risks of declining HGS in sarcopenia-risk populations.

This study has certain limitations. First, the cross-sectional design prevented the determination of causality. Second, the insufficiently detailed physical activity assessment may have led to a lack of correlation with muscle strength. Third, the subjective assessment of mood may have caused bias. Fourth, a limited number of chronic diseases were considered.

Nevertheless, the study provides an insight into the factors influencing HGS based on a variety of variables. It also supports the use of relative HGS as a simple screening tool and marker for sarcopenia in community-based healthcare settings and as an accessible strategy to improve overall health status.

## 5. Conclusions

The results of this study show that relative HGS is associated with physical, behavioral, and psychosocial factors in menopausal women. Identifying these factors can broaden our understanding of the potential risk of sarcopenia and provide insights into interventions that may contribute to the early detection and prevention of sarcopenia in middle-aged menopausal women. Furthermore, this information can be used as the basis for research and clinical practice guidelines to prevent sarcopenia in high-risk groups in the community, such as middle-aged menopausal women.

## Figures and Tables

**Table 1:** Sociodemographic and multifactorial characteristics of the participants (n = 2129).

Characteristics	Variables	Categories	n (Weighted %)	Mean ± SE
Sociodemographic	Age (y)	40–49	79 (4.5)	57.05 ± 0.11
		50–59	1281 (64.0)	
		60–64	769 (31.5)	
	Household income	High	670 (31.6)	
		Medium	1169 (55.5)	
		Low	290 (12.9)	
	Education	≥College ^§^	421 (19.6)	
		High school	775 (38.0)	
		≤Middle school ^¶^	933 (42.5)	
	Marital status	Living with	1709 (80.6)	
		Living without	420 (19.4)	
	Economic activity	Yes	1247 (58.4)	
		No	882 (41.6)	
Physical	BMI (kg/m^2^)	<18.5	57 (2.6)	23.95 ± 0.09
		18.5~24.9	1387 (65.3)	
		≥25	685 (32.1)	
	Abdominal obesity (cm)	Yes	607 (27.7)	80.49 ± 0.25
		No	1522 (72.3)	
	Comorbidity	Yes	458 (20.9)	
		No	1671 (79.1)	
Behavioral	Sedentary time (h)	≥8	923 (43.2)	7.10 ± 0.08
		<8	1206 (56.8)	
	Drinking (/month)	Yes	1309 (62.1)	
		No	820 (37.9)	
	Sleep duration (h)	<6	324 (15.1)	6.99 ± 0.03
		6~8	1477 (69.3)	
		>8	328 (15.6)	
Psychological	Depression	Yes	228 (11.2)	
		No	1901 (88.8)	
	Perceived stress	High	501 (24.0)	
		Low	1628 (76.0)	
	Self-rated health	Good	510 (23.6)	
		fair	1180 (55.7)	
		Poor	439 (20.7)	
Dependent variable	Absolute hand grip strength (kg)	<18 ≥18	162 (7.2%) 1967 (92.8%)	
	Relative hand grip strength (kg/BMI)			1.01 ± 0.01

BMI = body mass index; SE = standard error; ^§^ college or four-year university graduate above; ^¶^ primary school and middle school graduate.

**Table 2:** Difference in relative hand grip strength according to characteristics (n = 2129).

Characteristics	Variables	Categories	Mean ± SE	F	*p*
Sociodemographic	Age (y)	40–49	1.06 ± 0.03 ^b^	16.34	<0.001
		50–59	1.02 ± 0.01 ^b^		
		60–64	0.97 ± 0.01 ^a^		
	Household income	High	1.04 ± 0.01 ^c^	12.91	<0.001
		Medium	1.01 ± 0.01 ^b^		
		Low	0.95 ± 0.02 ^a^		
	Education	≥College	1.06 ± 0.01 ^b^	34.01	<0.001
		High school	1.04 ± 0.01 ^b^		
		≤Middle school	0.96 ± 0.01 ^a^		
	Marital status	Living with	1.02 ± 0.01	16.70	<0.001
		Living without	0.97 ± 0.01		
	Economic activity	Yes	1.01 ± 0.01	0.03	0.863
		No	1.01 ± 0.01		
Physical	BMI (kg/m^2^)	<18.5	1.20 ± 0.03 ^c^	202.89	<0.001
		18.5~24.9	1.06 ± 0.01 ^b^		
		≥25	0.88 ± 0.01 ^a^		
	Abdominal obesity	Yes	0.88 ± 0.01	301.40	<0.001
		No	1.06 ± 0.01		
	Comorbidity	Yes	0.95 ± 0.01	39.40	<0.001
		No	1.03 ± 0.01		
Behavioral	Sedentary time (h)	≥8	1.00 ± 0.01	1.11	0.293
		<8	1.01 ± 0.01		
	Drinking (/month)	Yes	1.02 ± 0.01	2.79	0.095
		No	1.00 ± 0.01		
	Sleep duration (h)	<6	1.00 ± 0.01	2.81	0.061
		6~8	1.02 ± 0.01		
		>8	0.98 ± 0.02		
Psychosocial	Depression	Yes	1.00 ± 0.02	0.41	0.520
		No	1.01 ± 0.01		
	Perceived stress	High	1.01 ± 0.01	0.03	0.869
		Low	1.01 ± 0.01		
	Self-rated health	Good	1.06 ± 0.01 ^c^	25.32	<0.001
		fair	1.01 ± 0.01 ^b^		
		Poor	0.94 ± 0.01 ^a^		

Different alphabets (^a,b,c^) indicate significant differences. SE = standard error.

**Table 3:** Factors influencing relative hand grip strength (n = 2129).

Characteristics	Variables	Categories	B	SE	95% CI	t	*p*
Socio-demographic	Age (y)	40–49	0.03	0.03	−0.02~0.08	1.27	0.203
		50–59	0.02	0.01	0.00~0.04	1.63	0.103
		60–64	(ref.)				
	Household income	High	0.02	0.02	−0.02~0.05	1.08	0.280
		Medium	0.03	0.02	0.00~0.06	1.75	0.080
		Low	(ref.)				
	Education	≥College	0.04	0.01	0.01~0.07	2.95	0.003
		High school	0.03	0.01	0.01~0.05	3.24	0.001
		≤Middle school	(ref.)				
	Marital status	Living with	0.04	0.01	0.01~0.06	2.91	0.004
		Living without	(ref.)				
Physical	Abdominal obesity	Yes	−0.16	0.01	−0.18~−0.14	−15.35	<0.001
		No	(ref.)				
	Comorbidity	Yes	−0.03	0.01	−0.05~−0.00	−2.23	0.026
		No	(ref.)				
Behavioral	Drinking (/month)	Yes	0.01	0.01	−0.01~0.03	1.29	0.199
		No	(ref.)				
	Sleep duration (h)	<6	0.01	0.01	−0.01~0.03	0.85	0.394
		>8	−0.03	0.01	−0.06~−0.00	−2.04	0.041
		6~8	(ref.)				
Psychological	Self-rated health	Good	0.06	0.01	0.03~0.09	4.31	<0.001
		fair	0.04	0.01	0.02~0.06	3.24	0.001
		Poor	(ref.)				

B = unstandardized regression; CI = confidence interval; SE = standard error; ref = reference.

## Data Availability

The authors declare that the data supporting the findings of this study are available within the paper.
